# The Power of Language in the Criteria for Restless Leg Syndrome: A Latin American Perspective

**DOI:** 10.1055/s-0046-1825530

**Published:** 2026-07-17

**Authors:** Maria Cecilia Lopes, Lucio Huebra, Gilmar Prado, Suzana Veiga Schonwald, Rosana Cardoso Alves, Sandra C Gonçalves Martinez, Carlos Maurício Oliveira de Almeida, Clélia Maria Ribeiro Franco, Raimundo Nonato Delgado Rodrigues, Paulo Afonso Mei

**Affiliations:** 1 Children's Institute, Hospital das Clínicas, University of São Paulo Medical School, São Paulo, Brazil; 2 Childhood and Adolescent Affective Disorders Program, Department and Institute of Psychiatry of University of São Paulo, São Paulo, Brazil; 3 Sleep Unit, Sirio-Libanes Hospital, São Paulo, SP, Brazil; 4 Department of Neurology, Universidade Federal de São Paulo, São Paulo, SP, Brazil.; 5 Unidade de Neurofisiologia Clínica, Serviço de Neurologia, Hospital de Clínicas de Porto Alegre, Porto Alegre, RS, Brazil; 6 Department of Sleep Medicine, Grupo Fleury, São Paulo, SP, Brazil; 7 Sleep Department, Brazilian Academy of Neurology (ABN), Department of Neurology, Hospital da Restauração, Recife, Pernambuco, Brazil; 8 Department of Neurology, Fundação Hospital Adriano Jorge, Manaus, Amazonas, Brazil; 9 Hospital Universitário Oswaldo Cruz, Universidade Federal de Pernambuco, Recife, PE, Brazil; 10 Faculdade de Medicina, Universidade de Brasília, Brasília, DF, Brazil; 11 Department of Medicine, Universidade Federal de São Carlos, Sleep Science Department, Academia Brasileira de Neurologia, São Paulo, SP, Brazil

**Keywords:** sleep related movement disorders, restless leg syndrome, Latin America

## Abstract

Adapting the American Academy of Sleep Medicine (AASM) Guidelines for restless legs syndrome (RLS) to Latin America is a complex challenge, given the region's 33 countries and vast socioeconomic diversity. Such an endeavor requires comprehensive data and consensus from a broader representation of these populations. There is a need to assess the effect of social vulnerability on the prevalence of symptoms of periodic leg movements that may be erroneously called RLS and mimic the primary nosological diagnosis for itself. We aim to discuss the Asociación Lationoamericana de Sueño's (Latin American Sleep Association, ALADS, in Spanish) position statement to contextualize the 2025 AASM clinical practice guidelines for the treatment of RLS and periodic limb movement disorder. Their intention of advocating for future systematic studies employing standardized criteria in Latin America. While the Scientific Committee for Sleep Disorders of the Brazilian Academy of Neurology partially endorses those opinions, offering some counterpoints concerning, especially, the following issues: 1) challenges to the reportedly wide variability in Latin American RLS prevalence, with apparently very high prevalences exceeding the expected values in some locations; 2) the importance of complementing clinical history with structured RLS diagnostic questionnaires; and 3) amendments to the proposed summary of treatment recommendations, particularly regarding Iron supplementation. Finally, the presence of comorbidities and RLS in epidemiological studies must be considered and data from Latin America can contribute to the discussion on global criteria applied worldwide for RLS that need to be revised according to regional, social, and cultural issues.

Could data collected in South America through Spanish-language interactions influence the diagnostic criteria for restless legs syndrome (RLS)? The 2025 paper by Parejo et al. 1 discusses RLS prevalence and treatment in Latin America, mentioning a particularly high prevalence in Colombia, especially in women, based on data published by Ruiz et al. 2 However, Ruiz's paper encompasses several sleep disorders, not only RLS, and the prevalence rates of RLS themselves vary across three different regions where data was surveyed. Discrepancies in prevalence compared to other countries in Latin American may be attributed to sleep fragmentation, poorly defined criteria for distinguishing periodic limb movements, and body movements from RLS. Moreover, regional and cultural factors impose challenges in understanding disease expression and its comorbidities.

Protective factors against iron loss, as well as issues related to diet, habits and lifestyle are paramount considerations. Is RLS more prevalent in socially vulnerable populations? Research conducted by large centers worldwide using well-defined inclusion criteria, excluding mimics and comorbidities, could provide a more homogeneous picture of RLS, hence allowing more effective pharmacological treatment.

Notably, the Latin American study of a rural population in a Brazilian region by Eckeli et al., 3 evaluated 1,228 adults. The lifetime prevalence of RLS was found to be 6.40%. Interestingly, a lower monthly family income (< $1,575 USD/month) was a protective factor. In implementation science, outcomes (feasibility, availability, and sustainability of diagnostic and therapeutic strategies) are conceptually and methodologically distinct from clinical efficacy, requiring specific forms of empirical evidence.

The discussion about iron intake in Latin America needs to be reviewed, 1 maintaining that ferritin behaves as an acute-phase inflammatory marker, and infectious processes can elevate its concentrations, compromising its isolated interpretation as a marker of iron stores. Nonetheless, there is no evidence to characterize Latin America as a region with permanent chronic inflammatory exposure at the population level, capable of systematically compromising the interpretation of ferritin in clinical practice.

Other factors may play a role in the discrepancy of the prevalence of RLS across Latin America, such as the possibility of overlap with attention deficit and hyperactivity disorder (ADHD), 4 potentially overestimating the prevalence of RLS in the Colombian cohort. Additionally, sleep breathing disorders may also increase the expression of ADHD and RLS symptoms. 5 Other studies without polysomnography used questionnaires to exclude individuals with clinical evidence of obstructive sleep apnea (OSA) in patients with RLS. 6

In our opinion, more information is necessary to explain the high prevalence of RLS in Latin America compared to other populations around the world, including whether the questionnaires used are validated in international consensuses, such as the clinical mnemonic “urge to move limbs; rest worsens symptoms; getting up or moving brings relief; and evening (or nighttime) worsening of symptoms” (URGE). In fact, in the data collected by Ruiz et al., 2 fulfilling two out of four URGE criteria was considered sufficient for RLS diagnosis. This implies that the prevalences reported in the Colombian study pertains to unspecific leg discomfort and not RLS per se.

The American Academy of Sleep Medicine (AASM) guidelines 7 cite a low prevalence of clinically significant RLS, defined as occurring at least 2 times/week in 2 to 3% of adults and 1% of pediatric population. From a regional perspective, the inclusion of broader data, especially from highly populated countries—such as those from Brazilian studies and consensus documents—could further strengthen the comprehensiveness of a guideline proposed for Latin America, given Brazil's sustained contribution to research on this syndrome. Scrutiny of data showing very high prevalences of RLS must be warranted and weighed against confounding factors and different entities—periodic limb movements (PLM) without RLS, anxiety, polyneuropathy, leg cramps, chronic pain, menopause, other sleep disorders, and secondary RLS. Epidemiological data must also be analyzed through the lens of social, racial, and ethnological factors.

On the matter of available medications, the claim that dopamine agonists remain widely used in Latin America primarily due to limited access to alternative drugs is not supported by real-world prescription data, systematic clinical practice surveys, or public policy analyses. In the absence of such data, this relationship remains hypothetical. 8

The Brazilian consensus reported a prevalence of 7% of RLS, and it was well discussed, regarding gender and age-related processes. Colombian data played a prominent role regarding to a specific point. Important global studies, following established protocols, often exclude patients with co-occurring sleep and clinical disorders, so some researchers probably are missing the background of the multimorbidity across lifespan. Eckeli et al. 3 suggest that patients with RLS have shorter lifespans, and they discuss the differences in prevalence rates in Latin America: 20% in Argentina, 6.4 to 15.7% in Brazil, 13% in Chile, 37.7% in Colombia, 2% in Ecuador, and 4.4 to 15.6% Mexico. The increase of the prevalence of RLS in Brazil showed in 2025 by Leite et al. 9 may be associated with the inclusion of individuals with higher frequencies of neuropsychiatric symptoms, poor sleep quality, higher BMI, and lower ferritin levels.

Beyond diagnostics and treatment of RLS, there is a need to evaluate its impact on general health. Over the past few decades, several cross-sectional and longitudinal studies have demonstrated an association between RLS, as well as PLM during sleep, with cardiovascular comorbidities and hypertension, 10 reinforcing the importance of systemic and long-term implications in the interpretation of prevalence data in different populations.

Therefore, the guidelines of a North-American institution (the AASM) 7 must be contextualized to Latin America, considering all the specific factors of each region, such as epidemiological, cultural, and economic factors, as well as healthcare access. The particularities of all countries must be considered and discussed by a greater plurality of specialists, to provide better guidance for all Latin American professionals. Ultimately, embracing sleep as both a protective factor and a sensor of health requires a united effort from all Latin Americans fighting for better sleep care.
